# The complete mitochondrial genome of *Ricania speculum* (Walker, 1851) (Hemiptera: Ricaniidae): investigation of intraspecific variations on mitochondrial genome

**DOI:** 10.1080/23802359.2020.1839366

**Published:** 2020-11-20

**Authors:** Hyobin Lee, Jonghyun Park, Hong Xi, Gwan-Seok Lee, Iksoo Kim, Jongsun Park, Wonhoon Lee

**Affiliations:** a Department of Plant Medicine, Gyeongsang National University, Jinju, Republic of Korea; b InfoBoss Inc., Seoul, Republic of Korea; c InfoBoss Research Center, Seoul, Republic of Korea; d Department of Agro-food Safety and Crop Protection, Crop Protection Division, National Institute of Agricultural Sciences, RDA, Wanju, Republic of Korea; e Department of Applied Biology, College of Agriculture and Life Sciences, Chonnam National University, Gwangju, Republic of Korea; f Institute of Agriculture and Life Science, Gyeongsang National University, Jinju, Republic of Korea

**Keywords:** Mitochondrial genome, *Ricania speculum*, Hemiptera, intraspecific variations, South Korea

## Abstract

We have determined a mitochondrial genome of *Ricania speculum* (Walker, 1851) collected in Jeollabuk-do, Republic of Korea. The circular mitogenome of *R. speculum* is 15,530 bp long which is shorter than that of the previous mitogenome of *R. speculum* by 199 bp. It includes 13 protein-coding genes, two ribosomal RNA genes, and 22 transfer RNAs. Intraspecific variation between two mitogenome of *R. speculum* was investigated: 171 SNPs and 18 INDELs were identified, presenting a high level of intraspecific variations on mitochondrial genome.

*Ricania speculum* (Walker, 1851) known as the black planthopper is an agricultural pest species in Asia, which has recently been introduced to Europe (Mazza et al. 2014). It is an polyphagous species that can feed on more than 60 different woody plant species belonging to 33 families (Mazza et al. 2014; Rossi and Lucchi 2015; Rossi et al. 2015) and currently distribute in China, Indonesia, Japan, Korea, Philippines, Taiwan, Vietnam, and Italy (Wilson et al. 2016). It is known that *Ricania japonica* contains yeast-like fungal symbiont which recycles uric acid secreted by the host with its own uricase (urate oxidase; EC1.7.3.3; Hongoh and Ishikawa 2000). Recent study of fungal mitochondrial genomes rescued from NGS raw reads of the *R. speculum* sample presented that it belonged to the family Ophicocordycipitaceae (Park, Xi, Park, Lee 2020). It indicates that NGS sequencing is an efficient method in detecting symbionts like previous studies (Bae et al. 2020; Park, Xi, Park, Nam, et al. 2020).

To investigate the intraspecific variations of *R. speculum* mitochondrial genome, we analyzed a mitogenome of *R. speculum* collected in Jungtosa Temple in Jeollabuk-do in Korea (35.6585365 N, 126.887853E; the specimen is stored in Gyeongsang National University, Korea, Accession number: 2018-HJ-56). DNA was extracted using DNeasy Blood & Tissue Kit (QIAGEN, Hilden, Germany). Sequencing library was constructed using Illumina TruSeq Nano DNA Library Preparation Kit (Illumina, San Diego, CA) following manufacturer’s recommendations with around 350-bp DNA fragments. Raw sequences obtained using Illumina HiSeqX (Macrogen, Korea) were filtered by Trimmomatic 0.33 (Bolger et al. 2014) and *de novo* assembled by Velvet 1.2.10 (Zerbino and Birney 2008), SOAPGapCloser 1.12 (Zhao et al. 2011), BWA 0.7.17 (Li 2013), and SAMtools 1.9 (Li et al. 2009) under the environment of Genome Information System (GeIS; http://geis.infoboss.co.kr/; Park et al., in preparation). Geneious R11 11.1.5 (Biomatters Ltd, Auckland, New Zealand) was used to annotate based on the previous mitogenome of *R. speculum* (NC_031369; Zhang et al. 2016).

The new *R. speculum* mitogenome (GenBank accession is MT834932) collected in Korea is 15,530 bp long, shorter than the previous *R. speculum* collected in China by 199 bp. The order and contents of 37 genes, 13 protein-coding genes (PCGs), 2 ribosomal RNAs (rRNAs), 22 transfer RNAs (tRNAs), were well conserved in *R. speculum* as in other hemipteran mitochondrial genomes.

Based on comparison of two *R. speculum* mitogenomes, we identified 171 SNPs and 18 INDELs of which total length is 233 bp. A number of these identified intraspecific variations were in the large side for insect mitochondrial genomes (e.g. 11–66 SNPs and 173–176 INDELs in two *Aphis gossypii* mitogenomes; Bae et al. 2020; Park et al. 2019), although these numbers may not be suitable for direct comparison as single mutations can cause different bases of variations. Eighty-six SNPs were in PCGs, 6 were in rRNAs, 5 were in tRNAs, and the rest were in intergenic spaces, especially the A–T rich control region. Twenty-five of the 86 SNPs in PCGs were non-synonymous while 52 were synonymous, which is similar to the ratio found in *Hipparchia autonoe* (Lepidoptera), 5:11, showing the total number of SNPs is irrelevant to the ratio of the SNPs (Lee et al. 2020). The remaining 9 SNPs were found in 3′ end of *nad2* and were unable to be determined either synonymous or not as preceding INDELs caused frame shifts.

The largest 175-bp INDEL was in the A-T rich control region created due to repeat region reduction against the previous mitogenome (NC_031369). Two and three INDELs were found near the 3′ ends of *nad1* and *nad2*, respectively, causing only minor frame shifts because they were only a few amino acids far from termination. Two INDELs, 2 bp and 1 bp long, found in *nad5*, however, were located near the 5′ end, though they also did not cause much mutations as frame shift caused by the first INDEL was recovered by the second one.

We constructed a Bayesian inference tree using MrBayes 3.2.6 (Huelsenbeck and Ronquist 2001) based on multiple sequence alignments of 16 planthopper mitogenomes, excluding the highly variable control regions, together with those of a cicada, *Magicicada tredecula* (NC_041656), and a frog hopper, *Philaenus spumarius* (NC_005944), as an outgroup. These sequences were aligned by MAFFT v7.450 (Katoh and Standley 2013). The tree was congruent with previous phylogenetic studies as family Ricaniidae was grouped with Flatidae, and then with Fulgoridae (Song and Liang 2013; Figure 1). All branches were supported with maximum posterior probability values (Figure 1).

**Figure 1.**
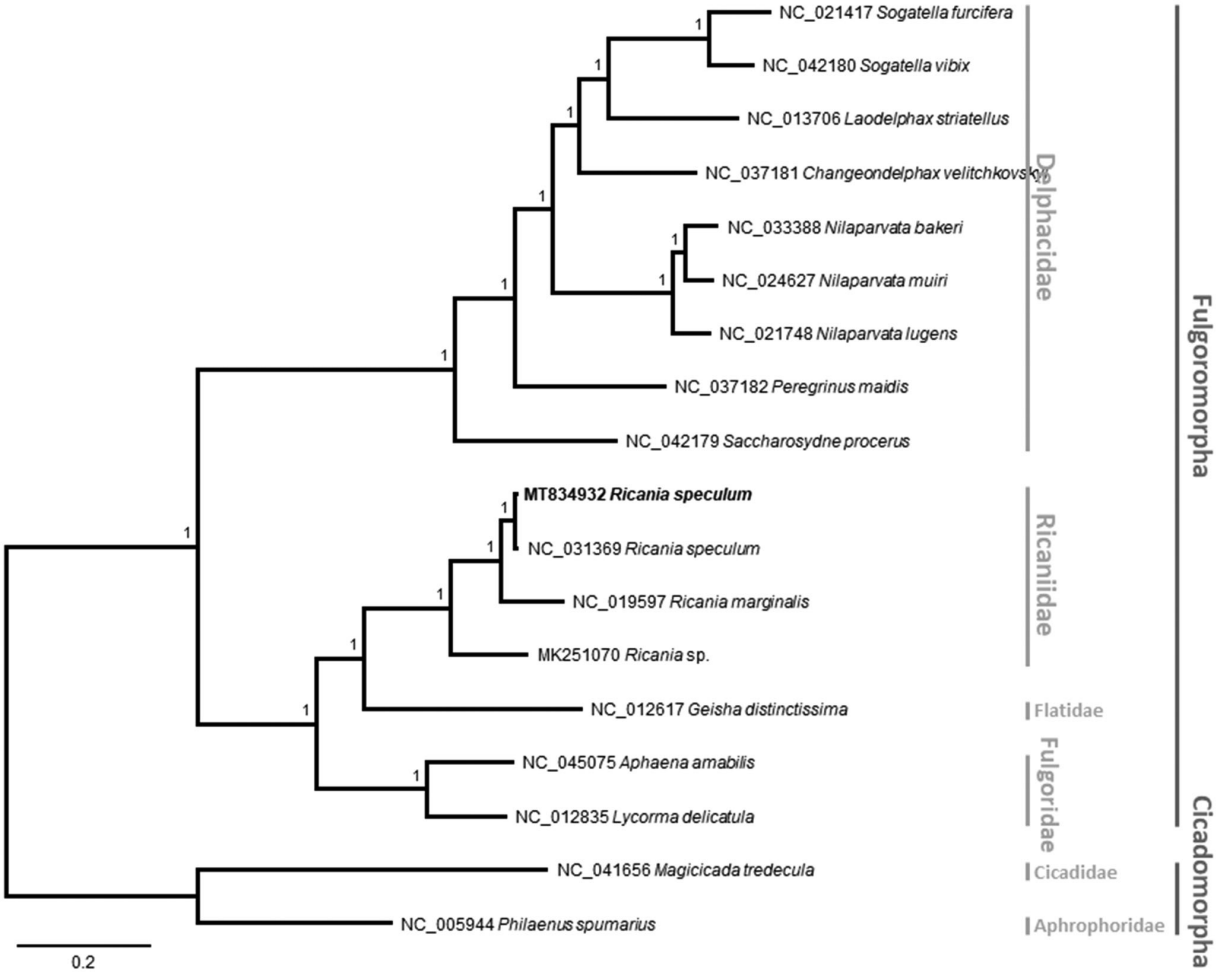
Bayesian inference (1,000,000 generations) phylogenetic tree of 16 Fulgoromorpha mitochondrial genomes: *Ricania speculum* (this study, MT834932)*, Ricania speculum* (NC_031369), *Ricania marginalis* (NC_019597), *Ricania* sp. (MK251070), *Geisha distinctissima* (NC_012617), *Aphaena amabilis* (NC_045075), *Lycorma delicatula* (NC_012835), *Sogatella furcifera* (NC_021417), *Sogatella vibix* (NC_042180), *Laodelphax striatellus* (NC_013706), *Changeondelphax velitchkovskyi* (NC_037181), *Nilaparvata bakeri* (NC_033388), *Nilaparvata muiri* (NC_024627), *Nilaparvata lugens* (NC_021748), *Peregrinus maidis* (NC_037182) and *Saccharosydne procerus* (NC_042179) and two out group species: *Magicicada tredecula* (NC_041656) and *Philaenus spumarius* (NC_005944). Numbers above branches indicate the posterior probability values of Bayesian inference. Family and infraorder names were displayed with gray bars in the right side of phylogenetic tree.

## Data Availability

Mitochondrial genome sequence can be accessed via accession number MT834932 in GenBank of NCBI at https://www.ncbi.nlm.nih.gov. The associated BioProject, SRA, and Bio-Sample numbers are PRJNA665776, SAMN16268313, and SRR12717137, respectively.
